# Skin and Neourethral Necrosis in Staged Hypospadias Repair

**DOI:** 10.21699/ajcr.v7i3.401

**Published:** 2016-06-15

**Authors:** Alireza Mirshemirani, Alireza Mahdavi, Mehdi Sarafi

**Affiliations:** 1 Pediatric Surgery Research Center, Shahid Beheshti University of Medial Sciences Tehran, Iran; 2 Pediatric Anesthesiology Department, Shahid Beheshti University of Medial Sciences Tehran Iran

**Keywords:** Hypospadias, Flap necrosis, Complication, Management

## Abstract

Complications in hypospadias surgery are not uncommon however penile skin or flap necrosis is rarely reported. Ischemia of the flap or graft is a major complication in two stage repair of hypospadias. A 2-year old boy with proximal penile hypospadias, operated earlier for chordee correction and urethral plate formation with a preputial flap, presented for stage 2 repair. Ten days after surgery patient developed skin and neourethral necrosis. Early debridement was done followed by coverage with scrotal flaps.

## INTRODUCTION

Hypospadias is an anomaly for which several procedures have been described. Technically flaps have a better blood supply than the grafts. Devascularization of the flap or graft is a major complication and reported incidence is 7%. [1-3] Necrosis may occur due to damage to vascular supply while raising the neourethral plate, vascular spasm, infection and tight pressure dressing. Herein a rare case of skin and flap necrosis during second stage repair of proximal penile hypospadias is reported.

## CASE REPORT

A 2-year old boy was admitted for second stage repair of proximal penile hypospadias with chordee. Six months earlier, chordee was corrected and neourethral plate was formed with vascularized preputial flap as first stage. Second stage was performed which included tubularization of the urethral plate and glanuloplasty. Patient was discharged after three days and urinary catheter removed at day 7 in outpatient department. Patient returned after three days with skin and neourethral necrosis (Fig. 1). In emergency, debridement was carried out under general anesthesia and a tube vesicostomy was performed (Fig 2). After one month vascular scrotal skin flap was applied to raw penile surface (Fig. 3). Urethroplasty with buccal mucosa planned after 6-12 months.

**Figure:**
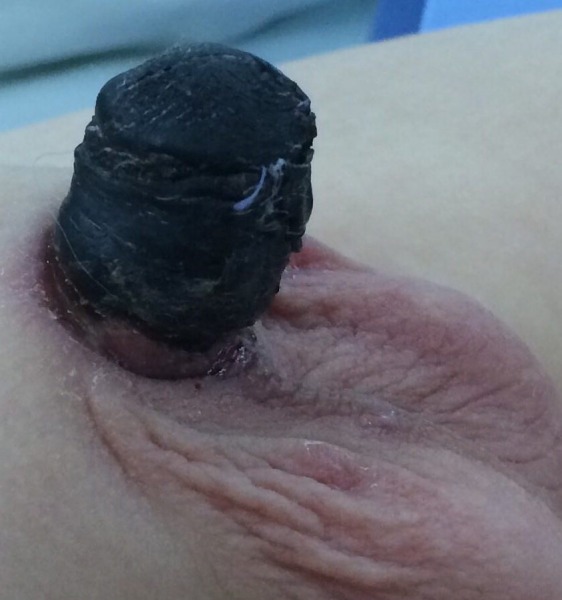
Figure 1:Necrotic penile and glans skin.

**Figure:**
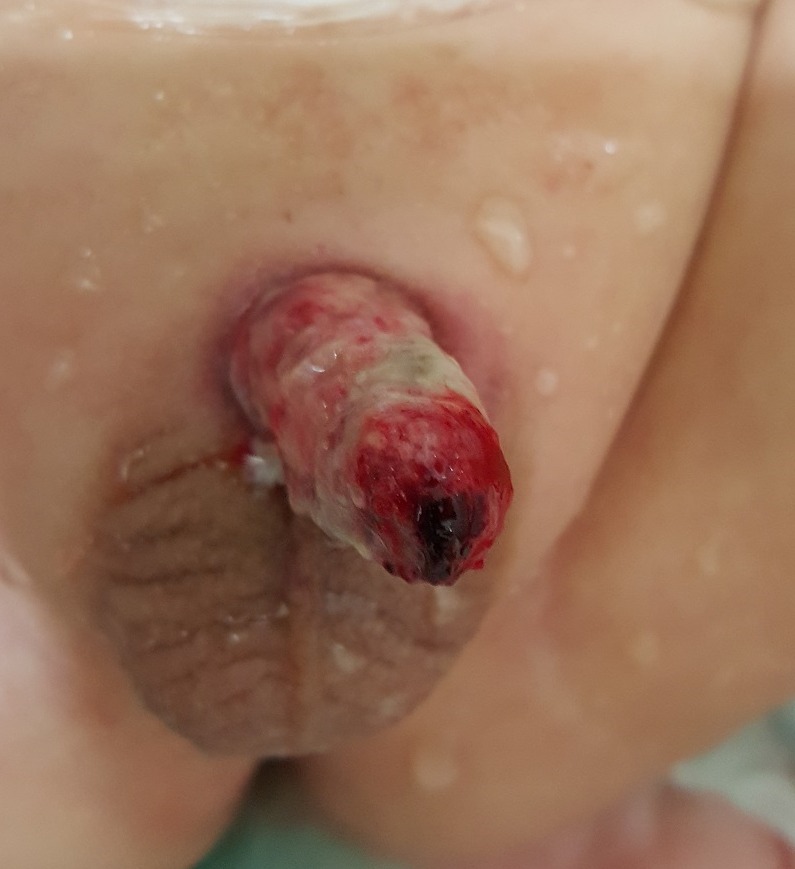
Figure 2:After debridement.

**Figure:**
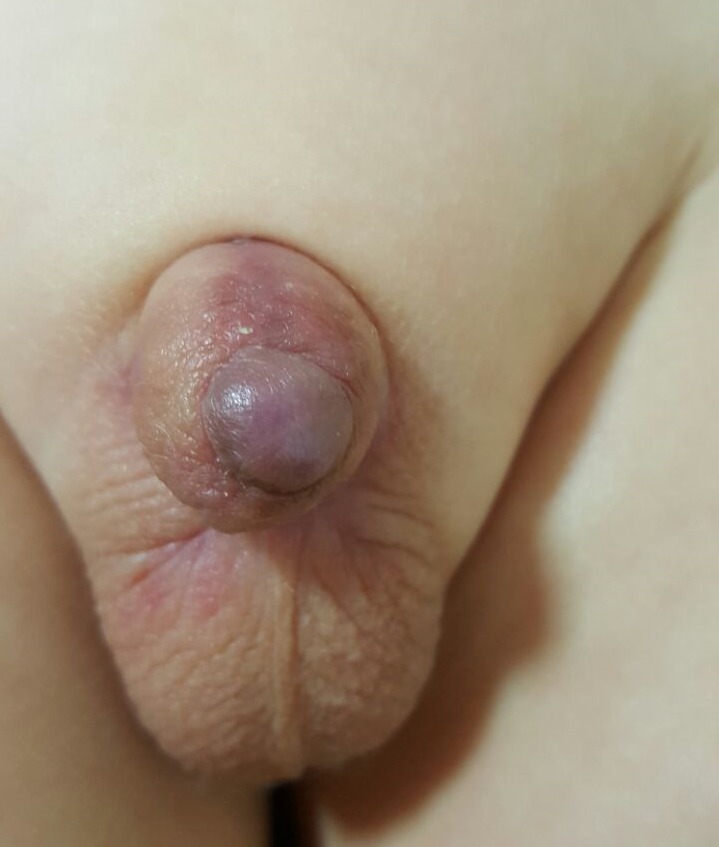
Figure 3:After scrotal flap cover.

## DISCUSSION

The incidence of complications in hypospadias repair ranges from 6 to 30%.[4, 5] This relates to the type of hypospadias, surgical technique, size of the penis, age of the child, and experience of the operating surgeon. Penile skin and flap necrosis is rarely reported. According to Bhat A et al the incidence was about 7%. [1] The necrosis of the skin may be superficial (mostly due to pressure dressing) and heal without permanent damage. In our case a tight dressing may be the cause as it was noted three days after catheter removal. Skin and flap necrosis can be prevented by good surgical technique, meticulous dissection, and appropriate use of dressings. If any part of graft or flap is devitalized, emergency debridement is needed, as done in our case.

## Footnotes

**Source of Support:** Nil

**Conflict of Interest:** None declared

